# Maternal depressive symptoms and stress during pregnancy as predictors of gestational age at birth and standardized body mass index from birth up to 2 years of age

**DOI:** 10.1186/s12884-021-04111-x

**Published:** 2021-09-18

**Authors:** Janina Eichler, Ricarda Schmidt, Tanja Poulain, Andreas Hiemisch, Wieland Kiess, Anja Hilbert

**Affiliations:** 1 Integrated Research and Treatment Center AdiposityDiseases, Behavioral Medicine Research Unit, Department of Psychosomatic Medicine and Psychotherapy, Leipzig University Medical Center, Semmelweisstrasse 10, 04103 Leipzig, Germany; 2 LIFE Child Leipzig Research Center for Civilization Diseases, Leipzig University, Philipp-Rosenthal-Strasse 27, 04103 Leipzig, Germany; 3 Department of Women and Child Health, Hospital for Children and Adolescents and Centre for Paediatric Research (CPL), Leipzig University, Liebigstrasse 20a, 04103 Leipzig, Germany

**Keywords:** Depression, Stress, Body mass index, Gestational age, Pregnancy, Children

## Abstract

**Background:**

While depressive symptoms and stress during pregnancy are known to affect gestational age and weight at birth, evidence on their impact on child anthropometric development in the long term remains limited, showing inconsistent effects. Importantly, previous research indicated a substantially stronger impact of categorically rather than dimensionally assessed mental health problems on birth outcomes and child development.

**Methods:**

The Patient Health Questionnaire was used to assess depressive symptoms and stress during the 2nd trimester of pregnancy dimensionally and categorically, with scores ≥10 indicating clinical significance. Gestational age at birth and BMI-SDS from birth up to 2 years of age were examined as dependent variables. Structural equation modeling was used to examine the prediction of birth outcomes and child anthropometry by mental health problems while controlling for multiple maternal and child characteristics in 322 mother-child dyads.

**Results:**

Dimensionally assessed mental health problems did not significantly predict birth outcomes. While categorical depressive symptoms significantly predicted a higher child BMI-SDS, categorical stress significantly predicted a lower gestational age at birth. Neither categorical nor dimensional mental health problems significantly predicted child BMI-SDS at 6, 12, and 24 months postpartum.

**Conclusions:**

Depressive symptoms and stress during pregnancy seem to differentially affect birth outcomes, and only if clinically relevant. The results implicate the importance to timely treat pregnant women that are greatly affected by mental health problems to potentially reduce adverse birth outcomes.

## Background

The prevalence of overweight and obesity in childhood is at a high level, with 38.2 million children up to 5 years of age having overweight or obesity worldwide [1]. Specifically, in Germany 15.4 and 5.9% of children and adolescents having overweight (age- and sex-specific percentile of the body mass index [BMI] > 90th) or obesity (BMI percentile >97th [2]). Crucially, longitudinal evidence showed that weight status is relatively stable from birth on [3]. For example, children who were large for gestational age at birth continuously had a higher BMI up to adolescence [3]. Particularly, lower birthweight and weight development during the first months of life were prospectively found to be key risk factors of central adiposity and weight-related comorbidities later in life [4]. Strikingly, recent evidence by Roy et al. showed that during the first 2 years of life a child’s BMI was a better predictor for early childhood obesity than the anthropometric standard measure weight-for-length used in most previous studies [5]. Therefore, to reduce the current epidemic in overweight and obesity in children, it is important to examine early risk factors of adverse weight development from birth onwards.

“Fetal programming” describes the influence of gestational risk factors on child development and long-term health [6]. In the context of fetal programming of weight development, adverse intrauterine conditions were predictive for inadequately low or high birthweight and subsequent weight problems in children [7]. Besides maternal (e.g., age, socio-economic status, BMI, gestational weight gain, and pregnancy intention [8–10]) and behavioral risk factors (e.g., smoking and malnutrition [11, 12]) mental health problems (e.g., depressive symptoms and stress [13–16]) were key conditions underlying fetal programming.

Concerning mental health problems during pregnancy, depressive symptoms showed high prevalence rates ranging between 9.2% for high and 19.2% for low and middle income countries [17]. Furthermore, up to 95% of pregnant women reported at least one psychosocial stressor (e.g., financial problems or health concerns [18]). Maternal depressive symptoms and stress during pregnancy were negatively associated with gestational age at birth, birthweight, and birth length [14, 15, 19–22] with 1st or 2nd trimester mental health problems showing strongest effects on birth outcomes [21]. Specifically, Accortt et al. systematically reviewed *N* = 83 studies and found that 12 of 50 studies detected that higher levels of depressive symptoms predict lower gestational age at birth; 14 of 33 studies showed that higher levels of depressive symptoms predict lower birthweight; and 57 studies observed non-significant associations [15]. Similarly, meta-analytical evidence demonstrated stronger effects of depressive symptoms during pregnancy on birthweight than on gestational age at birth [14]. These effects were especially observed for depressive symptoms exceeding cut-offs that indicate clinical significance [20, 23]. Concerning stress during pregnancy, only small but significant effects were found for stress prospectively predicting gestational age at birth and birthweight [19]. Notably, while studies on major life events, community-wide catastrophes, or chronic stressors during pregnancy consistently found a negative association with gestational age at birth and birthweight, studies on daily stressors and perceived stress during pregnancy yielded heterogeneous, partially lacking effects [16, 22, 23].

Besides programming of birth outcomes, mental health problems during pregnancy were found to influence child anthropometry in the long term [24]. However, there are only few prospective studies that examined the relation between depressive symptoms and stress during pregnancy and infant overweight and obesity, with mixed findings in samples of *n* = 181 to 12,931 mother-child dyads [25–33]. Specifically, three studies found depressive symptoms during pregnancy to positively predict child BMI up to 6 months as well as leg length, and central adiposity at 3 years of age [26–28]; six studies indicated depressive symptoms to negatively predict child body length at 3 months, weight(−for-age) and child BMI up to 3 years of age, and child height at age 4 [26–30, 32]; and three studies found no significant effects of depressive symptoms on child weight(−for-age) during the first year of life [25, 31, 33]. Evidence on the influence of stress during pregnancy on long-term child anthropometry is even weaker and focused solely on specific rather than chronic types of stress or stress of everyday life [28]. Maternal-reported family stress during the 2nd trimester significantly positively predicted BMI in *n* = 5328 children aged between 3 months and 4 years after controlling for a large amount of confounders [28]. Maternal experience of racial discrimination or bereavement during pregnancy significantly negatively predicted child BMI at 3 years of age (*n* = 539 [34]) or from 10 years of age onwards (*n* = 65,212 [13]), respectively.

Given the small or inconsistent effects of fetal programming through mental health problems – especially stress – during pregnancy on anthropometric outcomes of the child, more evidence is needed to understand how depressive symptoms and stress differentially affect gestational age at birth and child anthropometry from birth onwards. Because previous studies mostly neglected birth length, but only considered birthweight as primary outcome, nothing is known about the adequacy of anthropometrics, which are more precisely reflected in the BMI at birth. Therefore, the aim of this study was to examine the influence of depressive symptoms and stress (i.e., personal impairment due to stressful events of everyday life) during pregnancy on gestational age at birth and standardized child BMI from birth up to 2 years of age in a population-based prospective cohort study of German mother-child dyads while controlling for various maternal and child characteristics. Consistent with previous research showing stronger effects using categorical rather than dimensional measures of mental health problems [14], we hypothesized that depressive symptoms and stress exceeding categorical cut-offs that indicate clinical significance would be inversely related to birth outcomes, while dimensionally assessed mental health problems would have no significant effect. Based on inconsistent effects of depressive symptoms and stress on standardized child BMI from birth onwards, we did not expect significant predictions of child anthropometry at 6, 12, and 24 months postpartum by mental health problems.

## Method

### Participants and procedures

Data from the BIRTH cohort of the “Leipzig Research Center for Civilization Diseases (LIFE) Child Study” were used in the current study, collected between 2011 and 2017. This prospective population-based cohort study examined pregnant women and their children from fetal life until adulthood. All pregnant women in the area of Leipzig (Germany) had the possibility to participate in the LIFE Child Study; no exclusion criteria were given. Women were recruited through advertisement at medical institutions and via media. Interested pregnant women obtained detailed information about the study, data use, potential risks of participation, their right to withdraw from the study at any time without explanations or negative consequences, and provided informed written consent prior to participation. At the time of recruitment, monetary incentives (at maximum 20 Euro per child) and small presents for the child were given to compensate for participation. Well-trained research assistants conducted assessments at visits during the 2nd and 3rd pregnancy trimester, and at child birth until 20 years of age (for detailed information, see [35, 36]). The Ethics committee of the University of Leipzig, Germany (reg. no. 264–10-19042010) approved the study.

Because previous evidence suggests 1st or 2nd rather than 3rd trimester mental health problems to have a stronger impact on birth outcomes [21], 2nd trimester assessments were used in the current study. For the present study, the following inclusion criteria had to be met: (1) singleton pregnancy; valid data on (2) depressive symptoms or stress during the 2nd trimester; (3) child anthropometry and gestational age at birth; and (4) physical diseases during pregnancy. For the present analysis, (5) women were excluded when they self-reported diseases during pregnancy with potential influence on child anthropometry (i.e., vaginal bleeding during; hydramnios; oligohydramnios; placenta previa; placental insufficiency; anemia; urinary tract infection; gestational diabetes mellitus; hypo-; and hypertension). The eligible sample consisted of 463 mother-child dyads. A total of 139 mother-child dyads had to be excluded from the analyses due to not fulfilling inclusion criteria (1) to (5) as stated above: (1) *n* = 15, (2) *n* = 89, (3) *n* = 3, (4) *n* = 6, and (5) *n* = 26, resulting in a final sample of *N* = 324 mother-child dyads.

### Measures

#### Maternal characteristics

Age, marital status, and pregnancy intention (0 = *intended* or 1 = *unintended*) were assessed by self-report questionnaires at the 2nd trimester visit. The Winkler Index based on income, education, and occupation was used to classify the socio-economic status (SES) of the family with a range between 3 and 21 (3 ≤ low ≤8; 9 ≤ middle ≤14; 15 ≤ high ≤21 [37]). Pregravid height and weight were obtained through self-report questionnaires during the 2nd trimester visit. Gestational weight gain (kg) was computed by subtracting the pregravid weight from the objectively measured weight during the 2nd trimester visit via the calibrated scale “Seca 701” (Seca Gmbh & Co. KG, Germany).

Maternal depressive symptoms and stress were assessed by corresponding modules of the German version of the Patient Health Questionnaire (PHQ-D [38, 39]). The self-report PHQ was developed to screen for stress-related symptoms and mental disorders according to DSM-IV [40]. The 9-item depression module PHQ-9 was used to assess depressive symptoms during the last 2 weeks (0 = *not at all* to 3 = *nearly every day* [41]). In a previous study of *N* = 745 pregnant women, the PHQ-9 showed high specificity (85%) and sensitivity (84%) for a depression diagnosis [42]. The 10-item stress module refers to personal impairment due to stressful events (e.g., financial problems, health concerns, or stress at work) during the last 4 weeks (0 = *not impaired* to 2 = *strongly impaired*). Separate total scores were calculated for the depression and stress module by summing the items of each scale [38]. Scores of ≥10 indicate depressive symptoms or stress of clinical significance [39, 41]. To distinguish between pregnant women with and without symptoms of a major depressive disorder [41], and with and without moderate to high psychosocial stress scores we used these cut-offs in this study [39].

#### Child characteristics

Child sex (0 = *female*, 1 = *male*) and gestational age in weeks were determined at birth by physicians. Child weight and height were measured by physicians at various health check-ups including at 2–4 h (birth); 6–7 months (6 m); 10–12 months (12 m); and 21–24 months (24 m) after birth. As the child’s BMI was found to be a better predictor for early childhood obesity than weight-for-length measure, BMI was calculated (kg/m^2^ [5]) and transformed into sex- and age-specific BMI-standard deviation scores (BMI-SDS [43]).

### Statistical analysis

Data preparation included Shapiro-Wilks tests of normal distribution for all variables and checks for plausibility. Outlier analyses were conducted for gestational weight gain (*n* = 1 outlier), depressive symptoms (*n* = 6 outliers), and stress (*n* = 6 outliers), deleting normally or non-normally distributed data which were 3 standard deviations under or above the mean of each variable. Because of outliers in both mental health problems, *n* = 2 pregnant women had to be excluded from the analyses, resulting in a final sample of *N* = 322. SPSS for Windows (Version 22.0; SPSS, Inc., Chicago, IL) was used for descriptive statistical analyses.

**Fig. 1:**
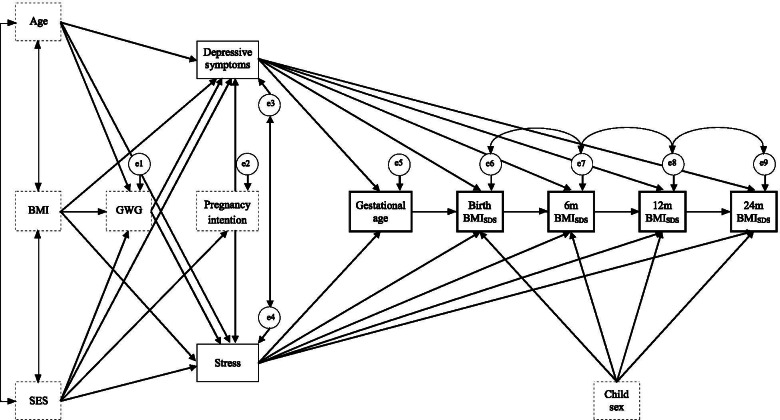
Structural equation model. Dotted squares display control variables; single bordered squares display independent predictor variables; double bordered squares display dependent variables; double-headed arrows display correlation coefficients between error terms; single-arrows display standardized regression coefficients. e = error term; Age = maternal age; BMI = pregravid body mass index (kg/m^2^); SES = socio-economic status; GWG = gestational weight gain; BMI_SDS_ = child body mass index standard deviation score at health check-ups at 2–4 h (Birth), 6–7 months (6 m), 10–12 months (12 m), and 21–24 months (24 m) after birth

Path analysis using structural equation modeling (SEM) was conducted to examine the influence of mental health problems on gestational age at birth and BMI-SDS at birth, 6 m, 12 m, and 24 m using Amos (Version 20.0; IBM SPSS, Inc., Chicago, IL, USA). Path analysis was run twice, once using dimensional (*Model 1*) and once using categorical mental health problems (0 = *women without depressive symptoms* or *stress of clinical significance*, 1 = *women with depressive symptoms* or *stress of clinical significance*; *Model 2*). According to existing evidence, maternal age, pregravid BMI, gestational weight gain, SES of the family, pregnancy intention, and child sex were entered as control variables in all analyses [9, 10, 44–46]. For handling missing data (for *n* of each variable, see Table 1), full information maximum likelihood method was used. Figure 1 displays the model of the path analyses. Relative strength of the examined relations was indicated by standardized βs. Model fit indices with recommended cut-offs were used to determine goodness of fit: χ^2^ (*p* > .05), root-mean-square error of approximation (RMSEA ≤ .05), comparative fit index (CFI ≥ .95), Tucker-Lewis index (TLI ≥ .95 [47]) and minimum discrepancy divided by its degrees of freedom (CMIN/df < 2). Statistical significance was set to a two-tailed *p* < .05.

**Table 1:** Prevalence rates, means, and standard deviations of study variables

*Variable*	*n*	*M* ± *SD*	*n* / %
**Mental health problems**			
Depressive symptoms (PHQ-9)	318		
Dimensional		4.89 ± 2.96	
Categorical (cut-off ≥10)			27 / 8.5%
Stress (PHQ-D)	318		
Dimensional		3.88 ± 2.93	
Categorical (cut-off ≥10)			18 / 5.7%
**Maternal characteristics**			
Pregravid BMI (kg/m^2^)	318	23.42 ± 4.41	
Gestational weight gain until 2nd trimester (kg)	282	6.88 ± 3.64	
Maternal age (years)	322	30.33 ± 4.17	
Winkler Index of the family	234	14.01 ± 3.91	
Low socio-economic status (3–8)			18 / 7.7%
Middle socio-economic status (9–14)			101 / 43.2%
High socio-economic status (15–21)			115 / 49.1%
Pregnancy intention (planned, yes)	292		243 / 83.2%
Parity (primigravid)	322		304 / 94.4%
**Child characteristics**			
Birth (health check-up at 2–4 h)	322		
BMI (kg/m^2^)		13.67 ± 1.30	
BMI-SDS		0.86 ± 1.07	
6 m (health check-up at 6–7 months)	271		
BMI (kg/m^2^)		16.71 ± 1.49	
BMI-SDS		0.12 ± 1.08	
12 m (health check-up at 10–12 months)	256		
BMI (kg/m^2^)		16.71 ± 1.46	
BMI-SDS		−0.03 ± 1.43	
24 m (health check-up at 21–24 months)	218		
BMI (kg/m^2^)		16.35 ± 1.35	
BMI-SDS		0.15 ± 0.97	
Gestational age at birth (weeks)	322	39.30 ± 1.48	

*Note. n =* number of pregnant women included in the model; *PHQ-9* Patient Health Questionnaire-Depression, *PHQ-D* Patient Health Questionnaire-German version, *BMI* body mass index, *SDS* standard deviation score

## Results

### Sample characteristics

Table 1 displays prevalence rates, means, and standard deviations of the measured study variables. Pregnant women during the 2nd trimester were 20 to 43 years old (*M* = 30.3, *SD* = 4.2) and reported a mean pregravid BMI (*n* = 318) of 23.4 ± 4.4 kg/m^2^ ranging from 17.2 to 48.4 kg/m^2^. Mean gestational weight gain until the 2nd trimester was 6.9 ± 3.6 kg ranging from −3.8 to 18.1 kg (*n* = 282). Socio-demographically, a major proportion of the pregnant women reported to be partnered (95.7%, *n* = 243 of 254), a middle (43.2%, *n* = 101 of 234) to high SES (49.1%, *n* = 115 of 234), had planned to become pregnant (83.2%, *n* = 243 of 292), and were primigravid (94.4%, *n* = 304 of 322). Concerning mental health problems, 8.5% (*n* = 27 of 318) and 5.7% (*n* = 18 of 318) of pregnant women exceeded the cut-offs for depressive symptoms and stress of clinical significance, respectively. Only a minor proportion of the sample exceeded the cut-offs for both depressive symptoms and stress (1.9%, *n* = 6 of 314). A minority of pregnant women gave birth preterm (4.3%, *n* = 14 of 322), with earliest birth at gestational week 31.

### Predictors of gestational age at birth and child anthropometry

Model fit indices for the path analyses for *Model 1*, χ^2^(39) = 51.770, *p* = .083; RMSEA = .032; CFI = .966; TLI = .920; CMHIN/df = 1.327, and *Model 2*, χ^2^(39) = 52.755, *p* = .070; RMSEA = .033; CFI = .954; TLI = .893; CMHIN/df = 1.353 were within the recommended cut-offs except the slightly lower TLIs, overall proving goodness of fit.

**Fig. 2:**
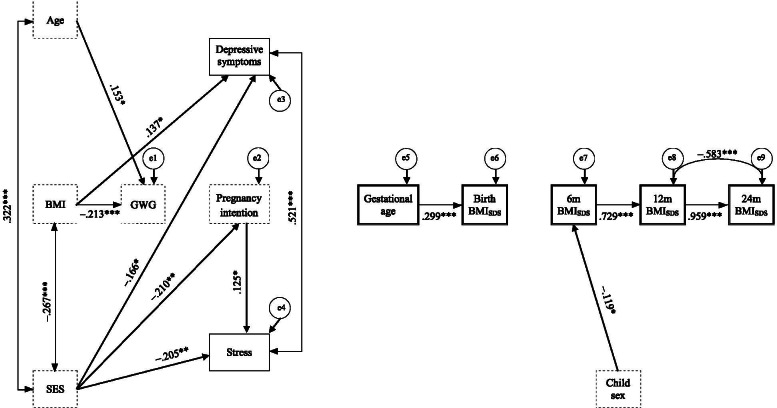
Structural equation *Model 1* using dimensional mental health problems to predict gestational age at birth and infant anthropometry. Dotted squares display control variables; single bordered squares display independent predictor variables; double bordered squares display dependent variables; double-headed arrows display correlation coefficients between error terms; single-headed arrows display standardized regression coefficients. e = error term; Age = maternal age; BMI = pregravid body mass index (kg/m^2^); SES = socio-economic status; GWG = gestational weight gain; BMI_SDS_ = child body mass index standard deviation score at health check-ups at 2–4 h (Birth), 6–7 months (6 m), 10–12 months (12 m), and 21–24 months (24 m) after birth. *** *p* < .001, ** *p* < .01, * *p* < .05

Figure 2 displays all significant paths of SEM for *Model 1*. Neither dimensional depressive symptoms nor stress during the 2nd trimester significantly predicted gestational age at birth or child anthropometry until 2 years of age (all *p*s ≥ .05).

**Fig. 3:**
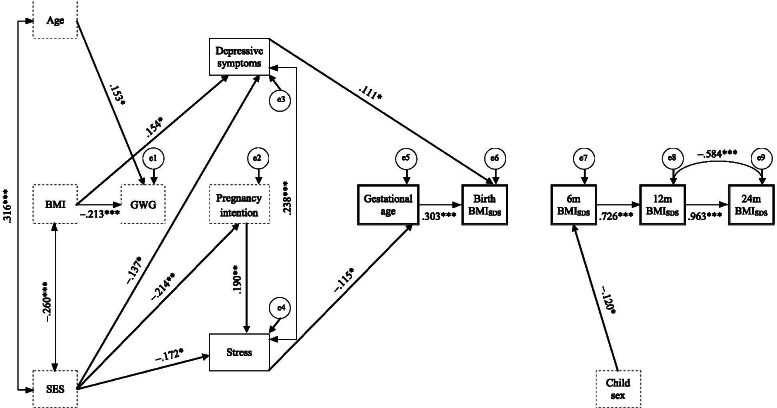
Structural equation *Model 2* using categorical mental health problems to predict gestational age at birth and infant anthropometry. Dashed squares display control variables; single bordered squares display independent predictor variables; double bordered squares display dependent variables; double-headed arrows display correlation coefficients between error terms; single-headed arrows display standardized regression coefficients. e = error term; Age = maternal age; BMI = pregravid body mass index (kg/m^2^); SES = socio-economic status; GWG = gestational weight gain; BMI_SDS_ = child body mass index standard deviation score at health check-ups at 2–4 h (Birth), 6–7 months (6 m), 10–12 months (12 m), and 21–24 months (24 m) after birth. *** *p* < .001, ** *p* < .01, * *p* < .05

Fig. 3 shows all significant paths of SEM for *Model 2*. Categorically, depressive symptoms of clinical significance significantly positively predicted BMI-SDS at birth (*p* = .044) and stress of clinical significance significantly negatively predicted gestational age at birth (*p* = .047).

## Discussion

To our knowledge, this is the first study that examined the influence of dimensional and categorical maternal depressive symptoms and stress during the 2nd pregnancy trimester on gestational age at birth and standardized child BMI from birth up to 2 years of age while controlling for multiple maternal and child characteristics in a population-based sample of German mother-child dyads. While dimensionally assessed depressive symptoms and stress were not associated with birth outcomes or child anthropometry at any age, depressive symptoms and stress of clinical significance significantly predicted a higher child BMI at birth and lower gestational age at birth, respectively.

In line with two previous systematic reviews showing larger effect sizes of categorically rather than dimensionally measured depressive symptoms or stress on gestational age and weight at birth [15, 22], mental health problems of clinical significance significantly predicted birth outcomes. The fact that depressive symptoms of clinical significance significantly predicted higher child BMI at birth, which is known to predict a continuously higher BMI up to adolescence [3], indicated that clinically relevant depressive symptoms during pregnancy may indirectly heighten the risk for overweight and obesity in children. Similarly, Ecklund-Flores et al. found a significant positive effect of depressive symptoms during the 3rd trimester on birthweight, while controlling for gestational age at birth [48]. Notably, most previous research demonstrating an inverse relation between depressive symptoms and birthweight did not control for gestational age [48], which may bias the results. Additionally, the majority of previous studies used birthweight rather than BMI at birth as their main outcome, with neglecting birth length. The fact that depressive symptoms of clinical significance predicted child BMI but not gestational age at birth is in line with previous evidence suggesting that depressive symptoms during pregnancy influence intrauterine growth, but do not cause pregnancy complications resulting in preterm or delayed birth [20, 23]. Stress of clinical significance negatively influenced the length of gestation, which is known to cause medical and social disabilities up to adulthood [49]. Though the effect size was small it was comparable to effect sizes found in previous research [19].

Dimensionally assessed mental health problems did not significantly predict birth outcomes. These findings were in line with expectations and previous research showing that 75% of 50 studies on predicting gestational age at birth and 47% of 33 studies on predicting child anthropometry at birth by depressive symptoms revealed non-significant results [15]. The lack of effect in predicting birth outcomes by dimensionally assessed stress is consistent with previous research focusing on stressors of everyday life during pregnancy suggesting that, stress due to major life events, community-wide catastrophes, and chronic stressors may be more relevant for child health outcomes [22]. In addition, as stress covers a broad construct with previous research merging symptoms of anxiety and distress with stress [19], different operationalization of stress may play a role when interpreting the findings.

The result that neither dimensionally nor categorically assessed mental health problems significantly predicted child BMI from 6 to 24 months postpartum is in line with expectations. It strengthened previous evidence showing no significant influence of depressive symptoms during pregnancy on child BMI in *n* = 168 to 12,391 children up to 3 years of age [25, 31, 33]. Studies reporting a significant positive or negative influence of depressive symptoms on child anthropometry found mainly small effects and revealed that chronic rather than episodic depressive symptoms during pregnancy might have a stronger impact on child anthropometry [26, 27, 29, 30, 32]. Concerning stress, our study adds unique evidence on the influence of chronic stressors of everyday life on child BMI to few previous studies on more specific types of stress (i.e., family stress, racial discrimination, and bereavement [13, 28, 34]). Taken together this suggests that major life events and chronic stressors may be more relevant in influencing child anthropometry than stressors of everyday life. According to our results and the inconsistency of previous results [28, 29, 33], it is likely that child characteristics (i.e., child sex and anthropometry) have a stronger impact on child BMI development than maternal mental health problems during pregnancy. Additionally, environmental factors related to mental health problems (e.g., family environment) might play a greater role in child BMI development than maternal mental health problems alone [28].

### Strengths, limitations, and future directions

A key strength of the present study is the prospectively assessed population-based sample of pregnant women excluding those with physical diseases during pregnancy with potential influence on child anthropometry. Mental health problems were assessed by an internationally established questionnaire showing good psychometric properties [38, 39, 42]. Confounding was minimized by examining depressive symptoms and stress simultaneously in the same model. Additional strengths include the objective multiple measurements of weight and height from birth up to 2 years of age. We controlled for many maternal and child characteristics known to affect gestational age at birth and child BMI from birth onwards. However, as a first limitation, we did not control for antidepressant use which might have biased the results because of its adverse effects on birth outcomes [50]. Second, only German women during the 2nd trimester of pregnancy were included reducing generalizability of the results to other nationalities or pregnancy trimesters, respectively. Third, the sample was selective in terms of a relatively high socio-economic status, which is a typical observation in population-based studies of pregnant women [51] and may be related to low levels of overweight and obesity and a low rate of medical complications in the present sample. In addition, the sample may be selective in terms of high pregnancy intention; bi-directional effects between pregnancy intention and health behavior may have a protective influence on women’s mental health problems. Thus, our results might not be generalizable to populations with a higher risk of experiencing mental health problems or comorbidities during pregnancy. Fourth, because self-report measurement of mental health problems may not accurately reflect health status, objective measurement (e.g., standardized interviews, physiological, and biochemical markers) would have been preferable, but is difficult to implement in a large population-based study. Finally, there was missing data on child BMI at 6, 12, and 24 months, but was corrected based on the full information maximum likelihood method.

The present study is a foundation for future research to further analyze the mechanisms contributing to adverse birth outcomes and child anthropometry from birth onwards. Because the effects of depressive symptoms and stress of clinical significance on birth outcomes and child BMI development up to 2 years of age were found to be small or non-significant, respectively, we recommend future research to assess mental health problems more than once during pregnancy (e.g., episodic versus chronic [28]) and in the postnatal period [27]; examine specific psychosocial symptoms more comprehensively (e.g., pregnancy-related concerns, family stress, and anxiety [22]); use objective assessment methods (e.g., blood examination); and consider other potentially confounding variables during pregnancy (e.g., maternal and behavioral risk factors [8, 11]) and in the perinatal period (e.g., non-breastfeeding, early introduction of solid foods [52]). Nevertheless, the results underline the importance of screening for mental health problems during pregnancy to immediately offer treatment (e.g., cognitive-behavioral therapy, group therapy, educational programs) to pregnant women affected by depressive symptoms and stress of clinical significance in order to potentially reduce the risk for adverse birth outcomes and postpartum depression [15].

## Conclusion

Pregnant women with depressive symptoms or stress of clinical significance had children with a higher BMI or gave birth at an earlier gestational age, respectively, than pregnant women without or only few depressive symptoms and stress during pregnancy. However, depressive symptoms and stress during pregnancy in otherwise healthy mothers did not lead to adverse child weight development from 6 months up to 2 years of age.

## Data Availability

The datasets used and/or analyzed during the current study are available from the corresponding author on reasonable request.

## Abbreviations

BMI: Body mass index
BMI-SDS: Body mass index standard deviation score
LIFE: Leipzig Research Center for Civilization Diseases
PHQ: Patient Health Questionnaire
pRR: Pooled relative risk
SEM: Structural equation modeling
SES: Socio-economic status
6 m: Health check-up at 6–7 months after birth
12 m: Health check-up at 10–12 months after birth
24 m: Health check-up at 21–24 months after birth
